# The face of equipoise - delivering a structured education programme within a randomized controlled trial: qualitative study

**DOI:** 10.1186/1745-6215-15-15

**Published:** 2014-01-09

**Authors:** Helen C Eborall, Helen M Dallosso, Heather Daly, Lorraine Martin-Stacey, Simon R Heller

**Affiliations:** 1 Social Science Applied to Healthcare Improvement Research (SAPPHIRE) Group, Department of Health Sciences, University of Leicester, Leicester, UK; 2 Leicester Diabetes Centre, University Hospitals of Leicester NHS Trust, Leicester, UK; 3 Academic Unit of Diabetes, Endocrinology and Metabolism, University of Sheffield, Sheffield, UK

**Keywords:** Behavioural intervention, Educator, Equipoise, Randomized controlled trial, Self-monitoring of blood glucose, Structured education, Type 2 diabetes

## Abstract

**Background:**

In trials of behavioural interventions, the individuals who deliver the intervention are in a position of key influence on the success of the trial. Their fidelity to the intervention is crucial. Yet little is understood about the experiences of this group of trial personnel. This study aimed to investigate the views and experiences of educators who delivered a structured education intervention to people with type 2 diabetes, which incorporated training in self-monitoring of either blood glucose (SMBG) or urine glucose (SMUG) as part of a randomized controlled trial (RCT).

**Methods:**

Educators’ views were explored through focus groups before and after training (*N* = 18) and approximately 1 year into the trial (*N* = 14), and semi-structured telephone interviews at approximately 2 years (*N* = 7). Analysis was based on the constant comparative method.

**Results:**

Educators held preferences regarding the intervention variants; thus, they were not in individual equipoise. Training raised awareness of preferences and their potential to impact on delivery. Educators were confident in their unbiased delivery, but acknowledged the challenges involved. Concealing their preferences was helped by a sense of professionalism, the patient-centred nature of the intervention, and concessions in the trial protocol (enabling participants to swap monitoring methods if needed). Commitment to unbiased delivery was explained through a desire for evidence-based knowledge in the contentious area of SMBG.

**Conclusions:**

The findings provide insight into a previously unexplored group of trial personnel - intervention deliverers in trials of behavioural interventions - which will be useful to those designing and running similar trials. Rather than individual equipoise, it is intervention deliverers’ awareness of personal preferences and their potential impact on the trial outcome that facilitates unbiased delivery. Further, awareness of community equipoise, the need for evidence, and relevance to the individual enhance commitment to the RCT.

**Trial registration:**

ISRCTN95696668

## Background

Equipoise can be defined as genuine uncertainty within the expert medical community regarding the comparative therapeutic merits of each arm in a trial [1]. A growing literature has demonstrated that trial participants have difficulties making sense of randomized controlled trial (RCT) procedures, random allocation and the principle of equipoise, and tend to hold preferences for certain treatment arms [2-7]. Clinician-researchers’ views also indicate varied levels of understanding of equipoise [8,9]. However, little is known about the views and experiences of those who deliver interventions in the RCT context (‘deliverers’), despite their influential position on participants’ adherence to the intervention and thus the outcome of the RCT.

In RCTs of pharmaceutical interventions, achieving equivalence in the delivery of the different arms is relatively straightforward; in a double-blind RCT, using a placebo that appears identical to a drug can reduce the potential for bias [10,11]. With interventions that cannot be blinded (for example, because the intervention cannot be disguised), minimizing variation in delivery presents more of a challenge [12,13]. This difficulty increases with the complexity of the intervention [14]. Randomized controlled trials of health and lifestyle educational interventions typically require an individual or team of individuals for delivery. These deliverers introduce the potential for variation in delivery, and thus a potential impact on trial outcomes [15,16].

Variation arising from different levels of skill and experience of deliverers can be reduced, to some extent, through training, and assessed through observation or recording of delivery [15,16]. Variation in deliverers’ behaviour, attitudes and beliefs, and the potential for these to distort the intervention delivery, are harder to address [17]. Furthermore, in a trial of two or more variants of a behavioural intervention, these must all be delivered in the same unbiased manner [15]. This raises the question of whether deliverers should be in a state of individual equipoise, that is, genuine uncertainty on their part regarding the comparative therapeutic merits of each arm in a RCT [1], or whether awareness of their own views is sufficient to avoid bringing bias to their delivery.

In this article, we explore the accounts of individuals delivering two variants of a structured education intervention as part of a multi-site cluster-randomized controlled trial. Of relevance is that one of the trial arms, which is education about, and training in, self-monitoring of blood glucose (SMBG) for self-management of type 2 diabetes, is the subject of much (and often emotive) debate; clinicians, nurses and patients have strong opinions on the matter; and thus, little individual equipoise (as demonstrated by a recent point-counterpoint feature in the journal *Diabetes Care*[18,19]). Furthermore, questions about the funding and cost-effectiveness of SMBG testing strips fuels the contentiousness of the debate and the strength of opinion [20]. We first situate our data in relation to two relevant areas of literature: role conflict and equipoise.

### Role conflict

The usual personnel involved in delivering, and communicating with participants in, clinical trials are doctors, nurses and allied healthcare professionals. In their everyday jobs, that is, when delivering care in a normal non-trial situation, they are expected to abide by a set of standards clearly defined by their respective professional bodies (such as the General Medical Council or the Nursing and Midwifery Council) with regard to their ethical conduct and roles. Some of the professional ethics and roles of a researcher may appear to be at odds with these.

In the case of the doctor-researcher, conflict may arise from different care responsibilities imbued by ‘normal’ doctor and researcher roles, whereby the level of care owed to trial participants differs from that owed to the doctor’s own patients [21,22]. One way of resolving this comes from the General Medical Council’s guidance for Good Medical Practice, which, while prioritizing the protection of research participants’ interests, emphasizes the need for participation in research for improving care for future patients and the wider population [23]. A second approach to resolving the tension borrows from the legal paradigm of ‘bailment’: a contractual agreement, whereby the custody of a patient’s care is temporarily transferred to the doctor-researcher, while remaining the overall responsibility of the patient’s usual doctor, allowing doctor-researchers to focus on the researcher side of their dual role [21,22]. While these approaches may help resolve conflict in theory, in practice, doctor-researchers have revealed unease and mixed understandings of the meaning of care in the trial context. For example, doctor-researchers have reported compensating for the altered duty of care by giving trial participants more attention and psychological support than expected in a usual clinical situation [22].

A similar role conflict can arise from the distinction between the set of values required for research and those required for nursing and midwifery, particularly pertaining to the consequences of research participation on confidentiality and trust in the nurse-patient relationship [24-26]. Unlike doctor-researchers, who may be more involved in the initiation and running of research trials, in the RCT context, research nurses are more likely to be involved in measuring, monitoring and caring for trial participants. Thus, their personal investment in the research question, and the importance they place on it, may also differ.

Randomized controlled trials of behavioural interventions, that is, educational or therapeutic interventions directed at behavioural or cognitive change, present fundamental differences from pharmacological or surgical RCTs, and raise different conflicts for personnel involved. On the one hand, the absence of pharmaceuticals, surgery or other medical treatment, may raise fewer concerns (for personnel) about potential adverse consequences of random allocation to a particular trial arm. On the other hand, conflict may arise from the requirement to deliver an intervention in an unbiased fashion while holding personal views about the research question. Such a conflict could potentially impact on the outcome of the trial through (intentional or unintentional) lack of fidelity in delivery of the intervention, perhaps by undermining the principle of equipoise.

### Equipoise and associated conflict

A central factor in the role conflicts discussed is the issue of a researcher’s personal views about, and emotional and intellectual investment in, the specific research question and trial. Not being in individual equipoise about the specific research question can impact on involvement in the trial in the first place, willingness to recruit patients, communication with patient participants, and delivery of the intervention [8,9,27,28]. Previous research has focused largely on the first two of these. For example, a study of clinicians recruiting to neonatal trials revealed that a lack of equipoise had a mixed impact on their comfort with recruitment. Some stated that they felt a moral obligation that they would not be able to take part if not in individual equipoise, whereas other (more experienced) clinicians recognized the need to accept collective or community equipoise, if evidence did not support their own personal view that one drug worked better than another [8].

A lack of equipoise might indeed raise clinicians’ interest in a trial. In a study of surgeons involved in a trial of invasive urodynamic testing, most were not in individual equipoise, as they believed in the benefits of the testing. Moreover, there was little evidence of community equipoise, that is, there was neither a majority of individuals reportedly undecided on the issue, nor a balance between those who regarded the treatment as beneficial and those who did not, as the majority considered it necessary [27]. However, the lack of evidence from trials at that point constituted formal community equipoise, and most surgeons interviewed recognized this. They were therefore willing to suspend the lack of either individual equipoise or informal community equipoise, and randomize their patients into such trials, with the intention that the results could provide the missing evidence that, they expected, would support their view and change others’ practice [27].

When involved in recruiting participants, researchers’ personal views can be unambiguous and influence potential participants’ decisions about participating (for example, see [7]), but little is known about the impact during intervention delivery. Useful insight comes from a study of research nurses involved in a trial comparing different ways of initiating intensive insulin therapy [28]. Nurses described their dilemma when the protocol required them to deliver different care from their usual practice, and prevented them from using their experience to provide individualized care. They queried the quality of the data informing the trial, and the lack of individual equipoise led some (particularly the more experienced nurses) to deviate from the trial protocol [28].

In this article, we explore the views and experiences of deliverers of a structured education intervention to people with type 2 diabetes incorporating training in either SMBG or self-monitoring of urine glucose (SMUG), as two arms of a RCT. Of particular interest, in this contentious area, was whether these intervention deliverers considered themselves to be in equipoise or not and, if not, how they felt this impacted upon their delivery.

## Methods

### Background: the DESMOND SMBG RCT

The DESMOND SMBG RCT (full title: ‘Does self-monitoring of blood glucose as opposed to urinalysis provide additional benefit to newly diagnosed individuals with type 2 diabetes receiving structured education?’; Trial registration: ISRCTN95696668) was a multi-site cluster RCT. It aimed to compare two self-monitoring strategies for people with newly diagnosed type 2 diabetes while controlling for the type and degree of education received [29]. Primarily, it aimed to ascertain whether there are equivalent changes in glycaemic control in participants allocated to SMBG or SMUG over 18 months when incorporated as an integral part of a comprehensive self-management structured education programme [30]. Full details of the intervention have been published in the RCT protocol paper [29].

The RCT was conducted in primary care in seven Primary Care Trusts (PCTs): public authorities in England which, at the time of this study, had responsibility for funding NHS services in a defined geographical area [31]. Participating PCTs needed to (a) be willing to cover prescribing costs for the strips required for the SMBG arm upfront (they were reimbursed at the end of the study), and (b) have a team of accredited DESMOND educators who were willing to participate. The PCTs and the educators within each PCT were therefore approached simultaneously. Within each PCT, participating practices were cluster-randomized, meaning that all enrolled participants in one practice were randomized to one arm of the trial, in order to avoid potential contamination. Practice nurses referred people who were newly diagnosed with type 2 diabetes and interested in participating to the local DESMOND team, who then checked willingness to participate and booked individuals onto a programme; educators were not usually involved in recruitment [29]. Educators were trained to deliver both variants of the study intervention, that is, the standard DESMOND programme with additional sessions on SMBG or SMUG. Full details of the trial and development of the interventions have been published [29]. The trial is due to report its findings in 2014.

In anticipation of the potential for educators to modify the intervention as specified during delivery, the RCT design incorporated specific educator training covering: evidence-based medicine, current evidence about self-monitoring, and the concept of equipoise. One element, ‘the ribbon task’, based on a bidirectional linear scale, [32] invited educators to indicate their preferences by choosing where to stand along a ribbon on the floor with the words ‘SMBG’ and ‘SMUG’ at each end and ‘neutral’ in the middle. Educators were invited to undertake this task individually and in a group setting on three occasions: before and after training, and one year later at a feedback session. Altogether, the training aimed to raise educators’ awareness of their own preferences and the potential impact of these on their delivery of the intervention, and equipped them with skills to prevent such an impact. Crucially, training was not designed to achieve a position where every educator was in individual equipoise regarding self-monitoring; rather, the focus was on educators exploring and acknowledging their personal preferences and their potential impact [29]. Finally, all educators had already been observed and assessed by DESMOND trainers as part of the standard DESMOND quality development system, in order to become accredited [33]. In addition, and in order to assess their fidelity to the intervention in the RCT, educators were observed and assessed delivering the additional sessions on SMBG or SMUG [29].

### Qualitative study with DESMOND educators

Ethical approval for this qualitative work was received (as part of approval for the RCT) from the Cambridgeshire Research Ethics Committee (07/H0304/129), and local research governance approval was obtained from participating PCTs.

All educators involved in the DESMOND SMBG RCT were invited to participate in focus group discussions and interviews held at different time points during the RCT. At each phase the invitation to participate was accompanied by information about the qualitative study. Phase 1: Focus group sessions were conducted at the beginning (part 1) and end (part 2) of two RCT training sessions. Phase 2: Follow-up focus group sessions were conducted at a feedback and training day approximately 1 year into the RCT (once educators had experience of delivering the interventions). All focus group sessions were 50 to 60 minutes long. Phase 3: Educators were invited to participate in a follow-up semi-structured individual telephone interview approximately 2 years after initial training. In phase 3, individual interviews were preferable to focus group discussions both because of the type of data of interest at this point, more in-depth reflections, and because it was convenient for the educators. Telephone interviews were 20 to 45 minutes long.

Flexible topic guides were used in the focus groups and interviews. Topics included: self-monitoring for type 2 diabetes, generally; the use of SMBG and SMUG, specifically; the RCT; reflections on the training; and experiences of delivering the interventions as part of the RCT. This paper draws on data generated across these themes, by exploring educators’ views pre-, mid- and post-delivery of the intervention, and focusing on whether they considered themselves to be equipoise or not, and why.

Informed consent was obtained prior to participation at each phase of the qualitative study. All focus groups and interviews were audio-recorded, transcribed and anonymized. Owing to anonymization, we were unable to compare educators’ individual views across all time points. Analysis was informed by the constant comparative approach [34]. Transcripts of phase 1 focus groups were read carefully, in order to develop preliminary codes, which were categorized into an initial coding framework and informed the development of the phase 2 topic guide. Transcripts of phase 2 focus groups were read carefully, in order to develop existing codes further and add new codes, and to inform the development of the phase 3 topic guide. The coding framework was finalized after phase 3; NVivo (QSR International) software was used to facilitate systematic coding of all data.

## Results

All 23 educators involved in the RCT participated in at least one phase of the qualitative study. All educators who attended one of the first two RCT training sessions (*N* = 18) agreed to participate in phase 1. All educators who attended the feedback day (*N* = 14) agreed to participate in phase 2 (9 of these had participated in phase 1). Seven educators agreed to participate in phase 3 (all of these had participated in at least one of the previous phases). Educators had a range of professional backgrounds (including: diabetes specialist nurse, dietician, podiatrist and practice nurse); all but one were female.

### Pre-RCT: lack of individual equipoise

Data from the phase 1 focus groups clearly demonstrated that educators held opinions about the two self-monitoring methods. At this point, there were four key threads running through educators’ discussions. First, the majority expressed a preference for SMBG, owing to its perceived accuracy, particularly compared with SMUG, which was seen as outdated. Second, there was acknowledgement that SMUG could be more appropriate in particular cases, for example, owing to its less technical nature, so it should not be ruled out altogether. Hence, the third and most dominant thread was an overwhelming preference to be able to allocate monitoring method on an individual basis according to each patient’s needs. Most qualified their opinions by referring to years of experience of working in diabetes care. The following extract is a typical example of how these first three threads were vocalized:

B2: …It depends on the patient really… I guess, as health professionals we’re used to dealing with [SMBG] now so probably more familiar with the use of that kind of system, how useful it can be; and if you want good, accurate results that are timely, if done the right way, then I guess blood glucose monitoring probably has the advantage. But it’s not for everybody, by any means, especially with diabetes type 2. It’s very hard to say which side of the fence I’m on…

B3: …If you’ve been around for a long time, you’ve seen urine testing come and go, seen it superseded by blood testing, and this is all well and wonderful [but] it feels like a backwards step sometimes…

B1: …We’ve flagged up times when it might be appropriate to recommend urine testing to people, which is something we’re not used to doing; and it wasn’t feeling like a backwards step but like another useful tool that could be used. (Phase 1, FG2, part 1)

However, a caveat to these views (and the fourth thread) was that appropriate training for patients, for example, through DESMOND, was required in how to use either method effectively, as demonstrated:

A4: I feel that the most important thing is that they’re educated to know what to do with the information, with their monitor, whether it be urine testing or blood glucose monitoring. They need to know what they should do with that information, to manage their diabetes. (Phase 1, FG1, part1)

Of more importance than the content of educators’ preferences was the evidence that they could not be considered to be in individual equipoise. Given their frontline role in delivering the intervention in the RCT, and their potential influence on its outcome, this presented a potential problem. Indeed, a couple of educators expressed concern that the allocation of participants to monitoring method required by the RCT might mean that some would be allocated to a method that would not suit them:

A2: …Some people are right for urine and some people are right for blood… and we’ve got our preconceived ideas [about] which… so are these people split down the middle…? (Phase 1, FG1, part 1)

Most educators reported that the RCT training had allowed them to discuss such concerns. The session about preferences and equipoise was reported as particularly useful; educators reflected on how the ‘ribbon task’ had raised their awareness of holding preferences, and had helped them realize the origins of their own views:

A5: I’ve had those views for a number of years, so it was nice to know from listening to everybody else why I felt those views ’cause you forget… they’re just there, aren’t they? (Phase 1, FG1, part 2)

The training appeared to have met the RCT team’s aim of conveying the importance to educators of concealing personal preferences during delivery:

B1: I don’t think my [preference] has changed but I think what it’s done has enhanced my awareness of not allowing those beliefs to impact on [my delivery]. (Phase 1, FG 2, part 2)

### RCT delivery: working to conceal preferences

In phase 1, educators expressed confidence in their ability to deliver both arms of the intervention without bias despite holding preferences or other concerns. Two factors helped facilitate this. First, the empowerment-based nature of the DESMOND programme meant that educators should never be didactic; rather by exploring participants’ views, they facilitate the development of self-management practice [30]. Second, distinguishing between one’s professional and one’s personal self helped reconcile the dilemma raised by awareness of personal preferences. The following extract demonstrates both factors:

B2: I would agree with that message that it’s OK to have an opinion; it doesn’t matter what that opinion is, as long as you don’t allow it into the study or when you’re in contact with a group.

B1: I think in true DESMOND style that shouldn’t happen; it should be how you’re thinking anyway, shouldn’t it? People are going to make their own decisions.

B3: …I think you have a professional head and a personal head and that made me think about that. I hadn’t thought of that before because I would say with my professional head on I was fairly neutral but then actually when I started talking about it, actually no; inside me I would prefer to be one or the other. But you recognize that as a professional you need to be more neutral. (Phase 1, FG2, part 2)

Indeed, in phases 2 and 3 when reflecting on their delivery, educators indicated how they drew upon their experience of professionalism (for example, from years of nursing) in order to help conceal their personal views:

E21: I think as a nurse you’ve always had to do things perhaps that you don’t want to do or don’t fancy doing so it’s part of your training… I’ve gotta do this, I’ve gotta put a smile on my face…

E16: I think it’s really dangerous and if people sort of approach a patient and bring their own negativity to that conversation and you can influence people in that way, so I think with experience and years of practice… you do tend to adapt and you do try to portray a very positive, very neutral… (Phase 2, FG1)

However, others acknowledged the hard mental work often involved in unbiased delivery. For example, some with a preference for SMBG reported the effort required to stay positive when participants allocated to SMUG returned to the second session having tried the method for a week with no success:

E5: I think delivering [SMUG] generally is harder… and you’re having to find resources within yourself to sort of teach it properly… because… they’re all coming back [testing] negative. So you’ve got to try and lead them to the point where they think yeah okay the urine’s negative but that doesn’t necessarily mean that my blood sugar’s normal and sometimes it’s hard to do that as a… you know, you really, really have to think about it. (Phase 2, FG2)

One reported frustration at not being able to train participants in the method that they believed would have suited them:

E2: There were moments when I thought a couple of members… were at a stage where they could have benefited from blood glucose testing… but that was just my own thoughts and I didn’t actually say to them… (Phase 3)

A couple of educators reported dealing with this situation by reminding themselves of the RCT protocol provision that participants could swap methods at the end of (or during) the RCT, if they struggled with their allocated monitoring method. In contrast, some educators reported finding their less preferred method easier to deliver; teaching SMUG was reported as less technical and less anxiety-inducing for participants who were worried about finger pricking.

E1: [Teaching SMUG] is so much easier practically… ’cause it took sort of takes less time. It took a lot of time to actually teach a group of people [SMBG]. (Phase 3)

### Commitment to non-biased delivery

Educators’ commitment to delivering the two arms with fidelity was highly salient throughout the data. When exploring this commitment further, several linked explanations emerged. First, many reported the importance of getting an evidence-based answer to a contentious issue; some mentioned their excitement at being involved with a trial that would provide such evidence.

E1: I think it’s a really important area. I do, and as far as, you know, we felt pleased that we could be part of it. There are so many papers written and whoever you’re talking to can come up with a different argument. That has never been done… as part of an education programme. (Phase 3)

There was a clear sense that educators felt there was a lot resting on the outcome of the RCT, most notably in terms of the potential influence on the prescribing decisions of the PCTs, which were referred to as having the final say regarding funding SMBG strips.

A5: At the end of the day, it’s driven by the PCT, isn’t it? The PCT say, ‘No,’ and therefore nobody gets it. (Phase 1, FG 1, part 1)

E2: I’ve already had two people from the commissioning board saying we’re waiting for [the results of] the DESMOND SMBG trial. (Phase 3)

Indeed some indicated their desire to communicate the findings to their PCT personally, if indeed the results support recommendation of SMBG:

E5: …and then can we go back to our Medicines Management people and say, ‘Right, we’ve got this piece of research and this shows that outcomes are better.’? (Phase 2, FG 2)

However, desire for the evidence was not limited to the wider context; a key driver behind their views was that educators were keen to learn the results themselves to inform them how to provide the best care to future patients.

E6: At the end of the day, we want to know… actually which is the best for patients. So you know we’ve got to be impartial, I suppose, in terms of how we come across with patients. (Phase 2, FG1)

## Discussion

This paper demonstrates that educators in the DESMOND SMBG RCT did hold preferences and opinions relating to (the two variants of) the intervention; they could not therefore be considered to be in individual equipoise. Training on evidence, equipoise and unbiased delivery raised educators’ awareness of personal preferences and the potential for these to impact on delivery. Educators were confident that they had achieved unbiased delivery, but acknowledged the challenges involved. Their perceived ability to conceal their own preferences was helped by a strong sense of professionalism, the patient-centred nature of the DESMOND programme, and concessions in the RCT protocol. Educators’ explanations for their commitment to unbiased delivery included a desire for evidence-based knowledge in this contentious area, to inform both local healthcare commissioners and their own future practice with patients.

It is not surprising that educators had personal opinions about the intervention variants; years of training, professional socialization and experience of working with individual patients with diabetes will have informed their views about effective management of the condition. However, awareness of the ongoing and contentious debate for and against the value of SMBG for people with type *2* diabetes meant that they understood that community equipoise existed, and thus recognized the need for evidence to answer this question. Furthermore, rather than this contentiousness and educators’ own views exacerbating the tensions arising from having to deliver both variants, it appears that these contributed to reinforcing educators’ views about the importance of neutral delivery.

The educators had not been involved in designing the RCT; their role was delivering the intervention according to the protocol. Previous research on role conflict and equipoise would therefore predict a possible dilemma in delivering the intervention based on random allocation, rather than on patient or educator choice [8,28]. Our data support this to an extent by demonstrating some educators’ frustration at not being able to tailor care to individual patient’s needs. However, contrary to previous research, educators reported confidence that they had not only adhered to protocol, but expressed a strong commitment to concealing their personal views, to ensure proper conduct in the RCT and thus produce much-needed evidence. Our data thus supports Hilton *et al*.’s argument [27] that believing in community equipoise can be sufficient in suspending one’s own individual equipoise. The surgeons in Hilton *et al*.’s study, however, were convinced of the validity of their own views and thus regarded the RCT as a vehicle for providing evidence to persuade the community at large of their opinion [27]. In contrast, the educators in our study appeared to be genuinely keen for the RCT to provide an evidence-based answer to the question, whatever the outcome, enabling them to deliver evidence-based care to their own future patients. Furthermore, in the event that the evidence supported recommending SMBG to this patient group, they anticipated also using the evidence to help persuade those in control of funding decisions in their PCTs.

The strong sense of professionalism and commitment indicated by educators when describing working to conceal their personal preferences echoes the idea of ‘active’ or ‘responsible’ uncertainty as a way of making sense of community equipoise, when not in individual equipoise [8]. Seeing the requirement to balance benefit against harm and engage with expert knowledge as part of one’s professional duty [8] appeared to help educators reconcile any distinctions between the values of the dual nurse-researcher role. Further, rather than educators’ professionalism from years of being a nurse (or other healthcare professional) leading to role conflict in the research situation, [22,24-26], educators appeared to draw upon professionalism as a useful resource in managing any tensions and their lack of individual equipoise.

This study was limited by an opt-in sample, and not all of the educators involved in phase 1 and 2 replied when invited for interview in phase 3. However, the data demonstrate that our sample held a complete range of views about the intervention variants. Furthermore, the prospective nature of the design allowed us to scrutinize views before and after educators had delivered the intervention variants in the trial. We acknowledge that the data are limited to educators’ reports of behaviour. Thus, we can only treat educators’ reports of unbiased delivery at face value. Educators’ delivery was, however, observed on at least one occasion by DESMOND trainers as part of the standard quality development process for all DESMOND educators [35].

### Implications

The finding that educators’ commitment to unbiased delivery was influenced by their desire for an evidence-based answer to an important question will be of interest to trialists. We argue that the importance of intervention deliverers ‘buying into’ the purpose of research highlights the value in training them in: the concept of equipoise, the potential impact of their preferences on the outcome of an RCT, the current state of community equipoise regarding the particular research question and the need for evidence. As discussed by others, the term ‘equipoise’ itself could help in such training of those less experienced in RCTs, by strengthening views and understandings of the challenging RCT situation; as the term is rarely used in everyday talk, it is less likely to cause confusion than words such as ‘uncertainty’ and ‘trial’, which may have several meanings outside the trial setting [7]. A key part of the training session in the current study was the emphasis that it was not aiming to convert each educator to a position of individual equipoise. Rather, the focus was on facilitating educators’ awareness of their individual views and their potential for these to impact intervention delivery, and providing them with the skills needed to prevent such an impact. Indeed integral to this was reassuring educators that not being in individual equipoise was acceptable, and that what mattered was awareness of one’s own preferences when in contact with participants. Our findings show that this approach was successful in raising the salience of the issue for deliverers, and suggest that it provided them with a useful resource in managing their own lack of equipoise when delivering the intervention. These are relevant and encouraging findings not just for those delivering an intervention in the RCT context, but for all personnel who have contact with participants, from the recruitment process through to trial closure.

## Conclusions

This study provides insight into a new group of personnel in the area of research on trial participation: deliverers of behavioural (educational) interventions. Rather than individual equipoise, intervention deliverers’ awareness of personal preferences and their potential impact on the trial outcome appeared to facilitate unbiased delivery if the intervention variants. Further, awareness of community equipoise, the need for evidence, and personal and professional relevance of the research area, enhanced deliverers’ reported commitment to the RCT. Incorporating training in these aspects can enhance deliverers’ commitment to the RCT and thus concealment of preferences during delivery.

### Ethical approval

Approval was received as part of the DESMOND SMBG RCT from the Cambridgeshire Research Ethics Committee (07/H0304/129) and local research governance approval was received from participating PCTs.

## Abbreviations

DESMOND: Diabetes education and self-management for ongoing and newly diagnosed; PCT: Primary care trust; RCT: Randomized controlled trial; SMBG: Self-monitoring of blood glucose; SMUG: Self-monitoring of urine glucose.

## Competing interests

HMD coordinated the DESMOND SMBG trial. HD and LMS designed the training for the DESMOND RCT educators. SRH is principal investigator of the DESMOND SMBG trial. He has served on advisory boards and given talks at meetings on behalf of Abbot and Lifescan, for which his institution has received fees. He also chaired the NHS Diabetes working group, which reported on SMBG in Type 2 diabetes in 2009. HCE declares that she has no competing interests.

## Authors’ contributions

HCE led the qualitative study, analyzed the data and drafted the manuscript and all revisions. HMD coordinated recruitment of qualitative study participants, helped draft the manuscript and contributed to revisions. SRH introduced the concept of equipoise into the protocol, and contributed to drafts and revisions of the manuscript. LMS and HD trained the educators in the principle of equipoise and contributed to drafts and revisions of the manuscript. All authors read and approved the final manuscript.
